# Supplementary material for: Bazedoxifene, a GP130 Inhibitor, Modulates EMT Signaling and Exhibits Antitumor Effects in HPV-Positive Cervical Cancer
Source: Int J Mol Sci. 2021 Aug 13;22(16):8693. doi: 10.3390/ijms22168693 (PMC8395523; doi:10.3390/ijms22168693)
Supplement: Supplementary file 1 [file ijms-22-08693-s001.zip › ijms-1331219-supplementary.pdf]

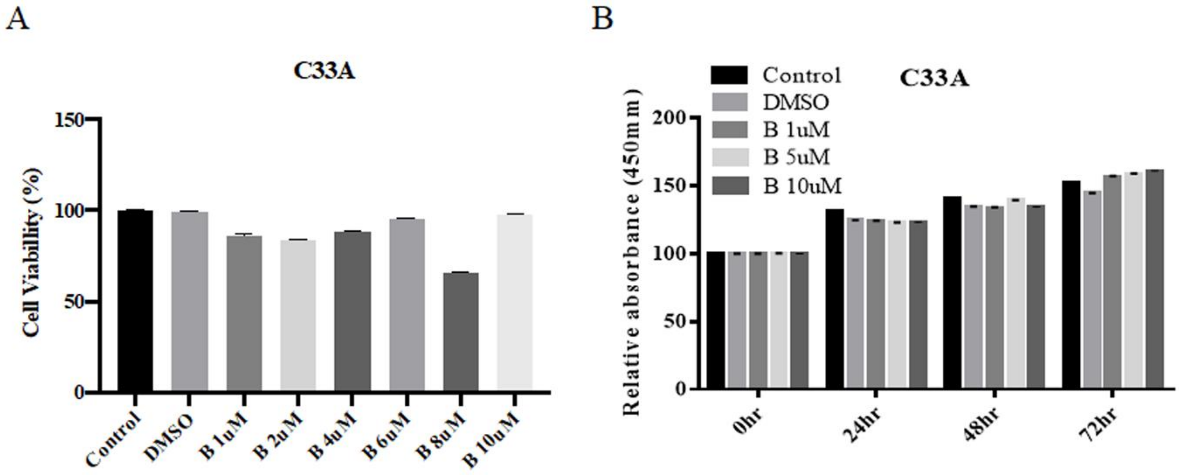

Figure S1. Concentration-dependent treatment of bazedoxifene in HPV-negative C33A cells showed no difference in cell viability. **(A)** C33A cells were treated with Bazedoxifene at the indicated concentrations in triplicate for 48h and processed for MTT assay to analyze cell viability. **(B)** C33A cells were treated with Bazedoxifene (1,5,10uM) in triplicate for 0, 24,48,72h and processed for CCK assay to analyze cell viability. B: Bazedoxifene
